# Open-source smartphone app and tools for measuring, quantifying, and visualizing technology use

**DOI:** 10.3758/s13428-021-01585-7

**Published:** 2021-06-03

**Authors:** Kristoffer Geyer, David A. Ellis, Heather Shaw, Brittany I. Davidson

**Affiliations:** 1 Department of Psychology, Lancaster University, Lancaster, UK; 2 School of Management, University of Bath, Bath, BA2 7AY UK; 3 Department of Engineering, University of Bristol, Bristol, UK

**Keywords:** Digital traces, Mobile software, Screen time, Smartphones, Technology use

## Abstract

Psychological science has spent many years attempting to understand the impact of new technology on people and society. However, the frequent use of self-report methods to quantify patterns of usage struggle to capture subtle nuances of human–computer interaction. This has become particularly problematic for devices like smartphones that are used frequently and for a variety of purposes. While commercial apps can provide an element of objectivity, these are ‘closed’ and cannot be adapted to deliver a researcher-focused ‘open’ platform that allows for straightforward replication. Therefore, we have developed an Android app that provides accurate, highly detailed, and customizable accounts of smartphone usage without compromising participants’ privacy. Further recommendations and code are provided to assist with data analysis. All source code, materials, and data are freely available (see links in supplementary materials section).

## Introduction

Human–computer interactions have become ubiquitous and continue to shape individuals and society (Ellis, 2019). For instance, many people in the general population will interact with their smartphone over 80 times a day in order access a variety of online services (Andrews et al., 2015; Ellis, 2019). As a result, the way in which individuals and groups use these technologies has provided new opportunities for research. Psychological science has specifically focused much of its efforts on understanding how technology use may impact health, social processes, and cognitive functioning (Ellis, 2020). For example, general smartphone use has been associated with a variety of negative outcomes including depression (Elhai et al., 2017), anxiety (Richardson et al., 2018), disrupted sleep (Rosen et al., 2016), cognitive impairment (Clayton et al., 2015), and poor academic performance (Lepp et al., 2015). This repeats a pattern of research priorities, which previously focused on the negative impacts of many other screen-based technologies, systematically moving from television and video games, to the Internet, and social media (Davidson et al., 2019; Rosen et al., 2014). In contrast, pre-registered studies have suggested that technologies, which were previously deemed problematic including social media and video games have negligible or positive associations with well-being (Johannes et al., 2020; Orben & Przybylski, 2019a).

The majority of research in this area shares a similar methodology when capturing technology usage. This typically relies on asking participants for a duration estimate or a qualitative reflection concerning their own experience rather than objectively measuring behavior from a device (Ellis, 2019). For example, requests for single duration estimates might ask: ‘how much time do you spend on your smartphone per day?’. Psychometric scales are also common and include a range of items that attempt to quantify ‘problematic’ or ‘addictive’ patterns of usage (Ellis, 2019). While such measurements are typical across social psychology, they have well-established limitations when attempting to describe behaviors or understand related psychological impacts (Baumeister et al., 2007; Doliński, 2018; Hinds & Joinson, 2019; Sassenberg & Ditrich, 2019). Single duration estimates are unable to capture the range of experiences provided by modern technology and survey instruments poorly correlate with a variety of objectively measured behaviors (Boase & Ling, 2013; Ellis et al., 2019). For example, picking up a smartphone is habitual and often occurs unconsciously throughout the day making it difficult to self-report accurately (Andrews et al., 2015; Ellis, 2019).

Researchers have suggested that the automated tracking of technology-related behaviors are valuable, but remain technically challenging to collect (Johannes et al., 2019; Orben & Przybylski, 2019b). However, a number of commercial apps can now quantify high-level aspects of smartphone usage, including total daily usage and number of pick-ups (Ellis et al., 2019). These include pre-installed tools such as, Digital Wellbeing (Google LLC, 2019) for Android systems, and Screen Time for iOS devices (Apple, 2019). While these apps can provide a more objective account of a person’s smartphone usage, they have several limitations. First, data are of a low resolution, and only provide daily metrics of usage. In order to answer specific research questions, hourly or minute-by-minute metrics are essential, however, the majority of third-party apps (e.g., *Moment, App Usage*) are, at the time of writing, unable to report the distribution of these smartphone use durations across multiple 24-h periods. Second, commercial apps cannot be re-configured to provide additional functionality. For example, there is often no way to export raw data, requiring participants to manually transfer statistics into survey responses (e.g., Ellis et al., 2019). As a consequence, commercial apps are ‘closed’ to the extent that researchers are unable to access source code, making it difficult to assess the reliability and validity of data collected.

In an attempt to circumvent some of these limitations, researchers have developed programming frameworks (e.g., *Funf in a Box* and *Aware*), which can facilitate the development of specific apps that could record technology-related behaviors (Aharony et al., 2011; Ferreira et al., 2015). However, these frameworks were predominantly designed to capture data from a variety of smartphone sensors. While the extensive APIs and associated libraries provide many data collection possibilities, this will always require significant development and customization before it becomes useful for researchers and safe for participants (Piwek et al., 2016). For example, a server will often be required to receive data remotely and researchers must implement sophisticated network protocols to maintain the security of data during transfer. Creating research-orientated apps from these frameworks therefore remains challenging for researchers who are unfamiliar with mobile app development and wish to ensure data succinctness.

To mitigate these issues and allow the research community to engage with objective methods, we present a customizable Android smartphone app – Usage Logger; and associated R scripts, Python notebooks (Jupyter), and websites (see supplementary materials). Together, these resources provide researchers with a straightforward way to capture a variety of smartphone usage behaviors. This includes the quantification of time spent on a device, specific app use, and notification activities.

Usage Logger timestamps every interaction the user has with their phone, which can generate a sophisticated understanding of general and specific technology usage. In addition, it can also extract historical data from the device, which addresses concerns of social desirability. As a result, the tools described here will help contribute to existing and new avenues of research. Specific research designs might consider, for example, if there are points in time where a participant’s usage is habitual or more entropic (Aledavood et al., 2018) or if usage was prompted by a notification or goal-directed. Given long-standing concerns regarding the impact of new technology, these resources will also allow researchers to better understand if certain patterns of usage have disproportionate associations with different psychological process and outcomes (Ellis, 2019). The rest of this article provides a comprehensive overview of the app and details how researchers can customize its operation, understand the data collected, and generate (or replicate) usage variables. All analysis scripts and associated software are freely available.

## Summary of architecture

The overall aim was to develop an app that will allow psychologists and others within the social sciences to conduct research that involves measuring smartphone technology interactions. The first step in the development of such a tool was to define the basic criteria that it needed to fulfil. For the aims of this project, these resources should: (a) provide open source code so that resources can be scrutinized and/or developed by other researchers; (b) record a variety of technology interactions while ensuring data succinctness (i.e., only data required is collected); (c) remain intuitive, practical, quick, and easy to use for groups of researchers who vary considerably in their computational literacy; and finally (d) keep data secure and provide privacy for research participants  with the opportunity to withdraw during any study (data remains on a device until participants wish to share it with researchers).

A variety of models have been proposed concerning software development lifecycles (Van Vliet, 2008). During development, we predominantly relied on a prototyping model because the system was developed alongside end users (researchers and participants) to improve each iteration of the software. As with related developments, Android was chosen as the initial development platform because it offered technical and methodological flexibility with the best cost–performance ratio (Geyer et al., 2019; Keil et al., 2020). Also, 80% of the worldwide market runs Android-related software (Keil et al., 2020).

**Fig. 1:**
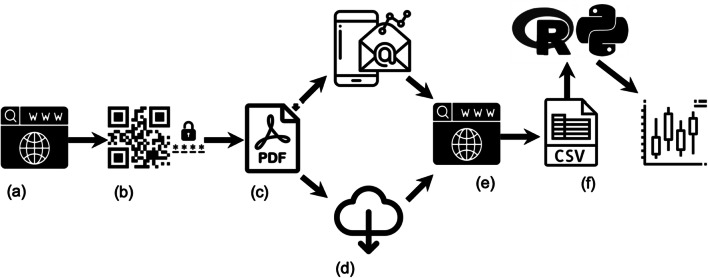
Overview of Usage Logger: (**a**) specification of configuration capabilities online; (**b**) a QR code is generated by the website and the app generates a secure password to encrypt all information and commences data collection; (**c**) post-data collection, .pdfs are generated; (**d**) these files can be exported via email, to another app or cloud service; (**e**) files can be decrypted via a second website; (**f**) this generates a .csv file, which can be processed using the provided R scripts and Python notebooks (Jupyter). All software and materials are open source and freely available in line with recommendations outlined by the UK Reproducibility Network (Turner et al. 2020)

The system consists of four major elements: a website to customize what data the smartphone app collects and/or retrieves, a Usage Logger app that enables data collection, a second website to assist with data processing, and a series of R scripts and Python notebooks (Jupyter) that contain live code to help with analysis (Fig. 1). In line with aim (a) concerning transparency, all third-party open-source libraries, which are essential to the functioning of the presented resources, are freely available (Table 1). As a result, researchers can take any single element, or combine materials as they wish and customize them as necessary. In the following sections, we describe each in detail.

**Table 1:** A list of third-party libraries used by Usage Logger and associated websites

Library name	Element	Link	Version	Function
JQuery	Customization website	https://blog.jqueryui.com/2015/03/jquery-ui-1-11-4/	1.11.4	Supports user interface
Canvas2Image	Customization website	https://github.com/hongru/canvas2image	NA	Converts QR code to png image
Qrcode	Customization website	https://davidshimjs.github.io/qrcodejs/	NA	Generates QR codes
Timber	App	https://github.com/JakeWharton/timber	4.7.1	Facilitates communication between app and developer
Dm77/barcodescanner	App	https://github.com/dm77/barcodescanner	1.9.13	Scans QR codes
Code Scanner	App	https://github.com/yuriy-budiyev/code-scanner	2.1.0	Scans QR codes post Android 8.1
Gson	App	https://github.com/google/gson	2.8.2	Converts Java objects to JSON
Armadillo Encrypted Shared Preferences	App	https://mvnrepository.com/artifact/at.favre.lib/armadillo/0.9.0	0.9.0	Encrypts data
SQLcipher	App	https://github.com/sqlcipher/android-database-sqlcipher	4.0.0	Constructs encrypted SQL databases
Jabit Spongy Cryptography	App	https://mvnrepository.com/artifact/ch.dissem.jabit/jabit-cryptography-spongy	2.0.4	Facilitates cryptographic calculations
iText	App	https://github.com/itext/itextpdf/releases	5.5.10	Constructs encrypted .pdfs
PDF.js	Data processing website	https://mozilla.github.io/pdf.js/	2.7.570	Supports data extraction from .pdfs

## Data sources and customization

A researcher will first have to specify via the customization website (see supplementary materials) what data will be collected by the app. In line with aim (b), this ensures that researchers are only collecting data that is essential to their research question where ethical approval has been received by a relevant committee. The customization website allows for three different data sources to be collected: *contextual*, *past usage*, and *continuous* usage. We outline these in more detail below. These summaries are also available as part of a companion website (see supplementary materials).

**Contextual** data provides a **snapshot** of information about software available on a device. Three sources of data can be extracted: installed apps, permissions requested, and responses to permission requests. Installed apps have previously been found to offer insights regarding personality (Xu et al., 2016). However, permissions requested and granted may also provide insights about a person’s attitudes towards privacy (Reinfelder et al., 2018). For example, a user with Facebook Messenger (Facebook, 2019) installed will be prompted to provide permissions that allow the app to access location sensing capabilities. This allows Messenger to send location updates to their friends and contacts. Responses to such requests also allow researchers to understand what data an associated app can access via the smartphones operating system. Using the contextual capability, the app generates a file containing this data called *“context.pdf*”.

**Continuous **logging collects information about multiple smartphone interactions as they occur **after** installation of Usage Logger. Researchers can decide what types of data should be collected including: when the phone is on/off, what apps are opened, if apps are added or removed (following installation or uninstallation), and when apps send notifications. Analysis of notifications for example, can help researchers differentiate between smartphone interactions that have been directed by an individual versus notifications that drive usage. The app will by default always document if a smartphone is restarted. All events captured by the app are UNIX timestamped and placed in a file called: “*continuous.pdf*”.

**Past usage** is based on how a participant has used their device previously, **before** installation of Usage Logger. Akin to an Internet browser history, these data are stored and maintained by Android devices. Usage Logger in this instance queries the appropriate database. Events documented include when a person turned their screen on/off, switched apps, switched their phone on/off, and how the operating system managed apps (Android, 2019a). Subsequent testing and reported validation suggests that this history typically includes multiple days of detailed data, but may vary between devices. This might be useful for a variety of studies as previous research suggests that only 5 days of retrospective data is sufficient to be representative of a person’s typical smartphone use (Wilcockson et al., 2018). It also avoids social desirability effects because unlike continuous logging, a participant cannot change their behavior before data is retrieved. Usage Logger returns a UNIX timestamp of when an event occurred, which app was involved, and what type of event occurred. This data is supplied in a file called “*usage.pdf*”.

## Order of collection

The customization website allows researchers to modify what data is collected by selecting specific sources. The order may be determined by dragging and ordering these sources accordingly. These decisions are likely to be driven by specific research questions. For instance, if a researcher wishes to review the impact of participants having data collected continuously, then they might collect 5 days of past usage and then contrast this with continuous data where a participant is aware that their usage is being recorded. Otherwise, if a researcher wants a higher degree of certainty regarding the collection of suitable data, they can collect several days of data prospectively and retrospectively query the same period afterwards. This will ensure that if  continuous data logging was paused at any point in time, higher-level missing data should be available via past usage as it relies on a different method of collection. It should be noted that participants have to trigger the switch from one data source to another (e.g., continuous logging to past usage). This feature was added to ensure that participants remain in complete control of data collected from their device (see consent and data security).

## Installation and operation

In line with aim (c), Usage Logger should be straightforward to use by researchers and participants. Following installation by participants, which can be accomplished via the Google Play Store (see supplementary materials), the app (~ 10.6 MB in size) will request permission to access the camera so it can scan QR codes. After researchers specify their requirements (via tick boxes), QR codes are generated by the customization website. These QR codes contain all the details Usage Logger needs to configure itself and perform the desired data collection. This method of configuration was selected because it allows researchers to request that participants download an identical app, which can be customized quickly and accurately without having to rely on further input from end users. This also removes the need to modify source code and, in turn, reduces the possibility of programming errors. QR codes also provide additional flexibility for researchers as they can be quickly made available to participants as part of physical research materials or placed online.

Once a QR code has been scanned, participants will encounter several dialog boxes containing information on: the purpose of the app, the type of data being collected, and security/data protection measures. A full version of the app’s privacy policy can also be made available to participants separately (see supplementary materials). A password is then generated and participants are asked to approve several additional permission requests (dependening on data sources). These include usage access, which allows the app to query a database maintained centrally by Android devices that contains historical records of previous use. In addition, notification access allows the app to detect notifications. After suitable permissions are provided, the app will commence data collection in line with what was assigned during customization.

## Data security and consent

Previous work by the authors has focused on the transmission of highly sensitive location data (Geyer et al., 2019). Following suit, the security and safety of participant’s data remains paramount (aim (d)). Usage Logger has been developed to ensure that participants have control over their data while it is collected. In the first instance, a password is generated to protect data files. Relying on participants to generate passwords to protect their own data is notoriously difficult (Nelson & Vu, 2010) as these are often vulnerable to cracking. Hence, we elected to generate passwords automatically. This utilizes ‘user-generated-randomness’ (Alimomeni, 2014), which relies on  the insignificant elements of participants actions to seed a random generator. Within Usage Logger, a UNIX timestamp is generated each time a participant confirms that they have read a message about how the app functions. These values are then stored in a random order. A value is randomly queried from this list and used to seed a random character generator to create a password. The random nature of these passwords makes them less vulnerable to dictionary attacks, which rely on databases of previously leaked passwords (Bellovin & Merritt, 1992). A variety of characters also make the password more resistant to brute-force attacks (Pliam, 2000). Participants do not have to remember this password but can recall it from the app at any time. Of course, this layer of security relies on the Android device itself remaining secure and participants not sharing their password. We therefore recommend that participants have a screen-lock or related system enabled on their device to prevent unauthorized access.

Data collected by Usage Logger is then stored in a SQLcipher database (Android, 2019b), which only Usage Logger can access provided normal security protocols are not extensively compromised. Accordingly, we would not recommend running Usage Logger on any device that has been rooted because this may undermine data security protocols. To protect users further, data is encrypted with a 256-bit Advanced Encryption Standards (AES) algorithm  (Zetetic, 2019). Data also remains secure when exported by being inserted into an AES 256-bit encrypted .pdf file after extraction  from the encrypted database. The .pdf is then supplied to the app (Android, 2019c), which can be attached to an e-mail or related service.  

The decision to develop an app with e-mail export capabilities ensures that researchers do not need to set up a cloud-based storage system. The source code could of course be modified or incorporated into any cloud-based development in the future. In the provided system, however, participants can straight forwardly remain in control of their data during collection. Presently, participants can withdraw before providing any data to researchers. In order for participants to pass their data onto the research team they must; install the app, read the instructions on how the app works, approve permissions, allow time to pass while data collection occurs, export data, and provide their password to a researcher.

This password handover process aims to strike a balance between providing functionality (so researchers can actually use the tools) and security (hence, data remains safe). It also has to be considered alongside how damaging a data breach might be for the individual. Several options are avaliable when securely transferring a password from participant to researcher, which are ordered from most to least secure. First, participants could simply read out their password to a researcher in a secure laboratory environment. Second, peer-to-peer encryption could be utilized using Telegram or similar apps, which sit outside an e-mail ecosystem, to transmit passwords (Barthelmäs et al., 2020). Finally, participants could send their password in a separate e-mail that does not include raw data.

At any point in this process, a participant can uninstall Usage Logger and all data will be erased. Researchers should request that participants uninstall the app after e-mailing data unless they wish to collect more data for personal use because continuous logging, if enabled, will resume. Beyond this, the graphical user interface (GUI) of the app has remained minimalistic to discourage excessive interaction and reduce the likelihood of demand characteristics impacting behavior. However, to ensure that participants are always aware that their behavior is being tracked during continuous logging, a notification will indicate that data collection is ongoing. This notification also  enhances the reliability of background data collection processes (Geyer et al., 2019).

## Reliability

Usage Logger has been designed to sustain continuous logging for substantial periods of time. The amount of data logged will be limited to the free space available, however the storage capacity of most modern smartphones is unlikely to impose any practical limits . However, some situations or actions will naturally impede data collection. For example, a participant might refuse or revoke permissions, force the app to close, or uninstall the app during the data collection process (Geyer et al., 2019). Usage Logger can generate crash reports (via .pdfs exported with raw data) that include details about the manufacturer/phone model, the section of code that caused the error, and a timestamp. Following a crash, the app will restart and ask participants if they are willing to continue. This feature is included to ensure that the app does not keep repeatedly crashing and instead requests that participants should contact the researchers or developers if problems persist.

## Validity

While it is not feasible to test software across every combination of the Android operating system and paired hardware, throughout development we wanted to ensure that Usage Logger can accurately collect data from the majority of smartphones in circulation. To confirm that Usage Logger is straight forward to use and collects data as intended, we engaged with three separate strands of validation that transitioned from qualitative logging and real-world user testing to the development of highly controlled, automated systems.The information gathered throughout supported subsequent development of the app and additional resources. 

### Log books

Throughout development and testing, researchers used pen-and-paper logbooks to ensure that actions performed on a given device were recorded by Usage Logger as expected. This process was repeated with each iteration of development to ensure functionality remained consistent.

### Real-world testing with participants

An earlier version of Usage Logger (Activity Logger) was tested in the field to ensure usability and validity. The resulting data from these tests are reported as part of Shaw et al. (2020). Using similar techniques, the app was set up to listen to three specific events: a phone being turned on, the screen being activated, and the screen being turned off. Participants who completed this earlier study (*N* = 46) reported no issues when installing or using the app and were asked to keep their phone switched on for the duration of the study (several days)^1^. Participants were also provided with visualizations of their usage patterns after taking part and were able to recognize repetitive patterns of daily usage. For example, when using their smartphone as an alarm clock, a regular marker of usage was repeated at the same time every day.

### Software validation

Finally, we conducted a series of automated validations with additional software. This involved running a separate app (Psych Validator – see supplementary materials), which programmatically causes a smartphone to execute specific functions  (e.g., open app, send/delete notification) or prompts a user to perform a particular action (e.g., turn on/off screen, un/install app). Psych Validator also documents the time at which these actions occur by listening in the same fashion as Usage Logger. To further validate some user-driven actions, Psych Validator performs additional inspections. For example, the number of apps available was checked for accuracy following each un/installation. Usage Logger was customized so that  past usage extraction occurred after continuous logging so both types of data could be assessed during  the validation.

## Method

### Procedure

We tested three popular Android smartphones from different manufacturers (Nokia, Huawei, and Google), which were running version 8 or later of the Android operating system. Usage Logger was installed, and permissions were enabled so that continuous logging would run as a background process. We then installed and started the validation app (Psych Validator).

This app requested a user perform a set number of actions: 20 screen on/off's, two identical app installations and two app un-installs. App events were initiated automatically: 10 notifications were pushed and removed, and a new app was opened 20 times. Data were then exported from Psych Validator and Usage Logger to the researcher’s e-mail. Time stamps of events prompted by the validator were aligned with recorded events from Usage Logger. The differences between average time stamps were then calculated.

### Results

These results confirm accurate functionality of the app to within a few seconds (Tables 2 and 3). All actions were correctly detected in the order they occured. However, not all attributes were captured at the exact time recorded by Psych Validator. Errors are reported in milliseconds.

**Table 2:** Descriptive statistics showing discrepancies (in milliseconds) between Usage Logger (continuous logging) and Psych Validator [Usage Logger timestamp-Psych Validator timestamp]

Device		Nokia		Huawei		Pixel	
Event	*n*	M	SD	M	SD	M	SD
Screen off	10	– 732.8	21.5	342.9	24.37	– 523.1	93.4
Screen on	10	– 476.2	9.5	342.1	15.39	– 502.2	157.4
App opened	20	563.6	406.9	– 557.4	334.1	523.6	253.1
Notification generated	10	114.9	22.1	184.6	10.58	332.9	589.3
Notification removed	10	12.5	14.7	227.8	13.17	34.5	91.5
App installed	2	– 665	1652	– 636.5	121.5	– 2302.5	1371.5
App uninstalled	2	2182	1117	907	19	– 1578.5	1368.5

*M* = mean, *SD* = standard deviation

**Table 3:** Descriptive statistics for discrepancies (in milliseconds) between Usage Logger (past usage) and Psych Validator [Usage Logger timestamp-Psych Validator timestamp]

Device		Nokia		Huawei		Pixel	
Event	*n*	M	SD	M	SD	M	SD
Screen off	10	1666.4	1662.8	– 1026.4	28.7	– 2535.1	2145.2
Screen on	10	– 313.9	1066.8	– 617.2	46.3	1217.9	1118.2
App opened	20	316	148.9	57.4	18	– 470.6	1101.3

*M* = mean, *SD* = standard deviation

It appears that some actions were actually detected up to a few seconds before they occurred. In these instances, Usage Logger appears to be predicting the future. Of course, this is not possible, but a consequence of how Android and other operating systems run multiple programs across physical processors (Novac et al., 2017). While it appears that multiple programs are operating in unison, this is an illusion. Android maintains a list of all programs currently running and swaps between them quickly so that users perceive them to be running simultaneously. Programs swap in and out of being executed in the order of every few milliseconds, but the order in which programs are swapped in and out of being executed will vary depending on a variety of other factors including task priority, which will be determined based on other background and foreground processes (He et al., 2019).

When an event occurs that Usage Logger records, it is possible that Android will let Usage Logger know the event has happened before it lets another app deal with the event itself. For example, when a "screen on" event occurs, the first part of Android to know that "the screen is going to be turned on" is called the Kernel. The Kernel does two things with this information: 1) it adds the "screen on" event to the list of logged events which need to be processed and 2) it adds the command "turn on the screen" to another list of processes that need to be actioned in the near future. Having logged what has occurred, the Kernel then decides what to do next. It could choose to actually turn on the screen, or to swap in the Usage Logger app (which will record the event) or do something else entirely. There are no guarantees about what happens first and so the "screen on" event could be recorded before the screen actually turns on, or vice versa. This effect also varies between devices. However, this variance operates in a fashion that is unlikely to affect the results of most investigations. If we reduced the precision of some  timing measurements, the effect would disappear completely. Regardless, we feel it is important to acknowledge these limitations here as part of a complete validation.

Overall, the quality of the data is high and suitable for the majority of research purposes that, depending on the measures used, do not require second or millisecond timing accuracy for individual events. We recognize that this specific form of validation represents a very small number of smartphones running popular versions of the Android operating system. Researchers can of course conduct their own validations as the source code of Psych Validator is freely available (see supplementary materials). Alternatively, participants could, at random intervals, report what they were last using their device for via ecological momentary assessment, which could be compared with objective logs. However, this again relies on participants correctly remembering individual technology interactions, which previous research suggests is far from accurate (Andrews et al., 2015).

## Data processing and analysis

After reliable and valid data have been collected, a second website (see supplementary materials) has been developed to help researchers decrypt participant data easily. These tools are also open source and can be developed further by other researchers. JavaScript, run from within the provided website enables the decrypting of .pdfs using pdf.js (Mozilla 2019), which allows for the rendering of text while retaining its position. This helps produce an easily interpretable .csv file. Alternatively, data can be decrypted without this website for example, by using the pdfTools package in R (Ooms, 2020) however, some tools can occasionally struggle to separate different cells in a .pdf file.

The remainder of this section will walk through the process of analyzing example data provided in the supplementary materials. The data will be processed, informative variables computed, and a simple data visualization generated. The included Python notebook (Jupyter) and R scripts replicate these calculations and visualizations.

## Data processing

Many data processing decisions that relate to collected data will be dependent on specific research questions. First, the researcher must choose which events are relevant. These can include: app moved to foreground, app moved to background, user interaction occurred, etc. (Android, 2019a). Events that participants have no control over (e.g., configuration changes, flush to disk events) are also recorded. We have left space for researchers to  determine events of interest in the included scripts. A second stage of processing involves removing any duplications (if required). Duplications are more likely to appear in retrospective extractions  (not during continuous logging) where the Android operating system is responsible for developing and curating the dataset. However, if the same event was documented as occurring twice within a few milliseconds, we can be certain that the duplication is a simple double-count issue. We also note that in most other instances, repetitive behavior is common for the majority of smartphone users in the general population (Shaw et al., 2020). Following this processing, single or multiple events can be extracted into a data frame. Researchers can also remove (or flag) events that appear to be the result of a participant simply leaving their phone on rather than actively using their device. For instance, a clock app appearing in the foreground for multiple hours may be due to a participant having their device’s screen set to remain on during charging (note: duration of event must be established before this processing can occur). After cleaning past usage data specifically, it should be in a relatively similar format as continuous data and can be utilized to compute identical variables.

## Establishing informative variables

The sheer number of potential variables is beyond the scope of this article. However, the supplementary code extracts a commonly referred to, but rarely measured metric, specifically, total time spent using a smartphone. Unix time stamps can be compared between a ‘Screen On’ event and ‘Screen Off’ event to calculate the duration of smartphone use. By selecting specific time frames, a variety of descriptive statistics can quantify hourly, daily, and weekly use. Similarly, establishing which apps a participant has spent more time using can be quantified by extracting app event logs, calculating the time differences between consecutive events and summing those durations independently.

There are several other are instances where it may be advantageous to combine contextual information with usage logging. For example, while contextual data provides a single snapshot of installed apps and associated permissions, this process can be repeated or combined with continuous logging in order to better understand privacy and security from the perspective of apps or participants (Ellis, 2020). Specifically, the extent to which participants approach permissions across all or specific apps installed on the phone can be explored dynamically over time.

### Visualization

**Fig. 2:**
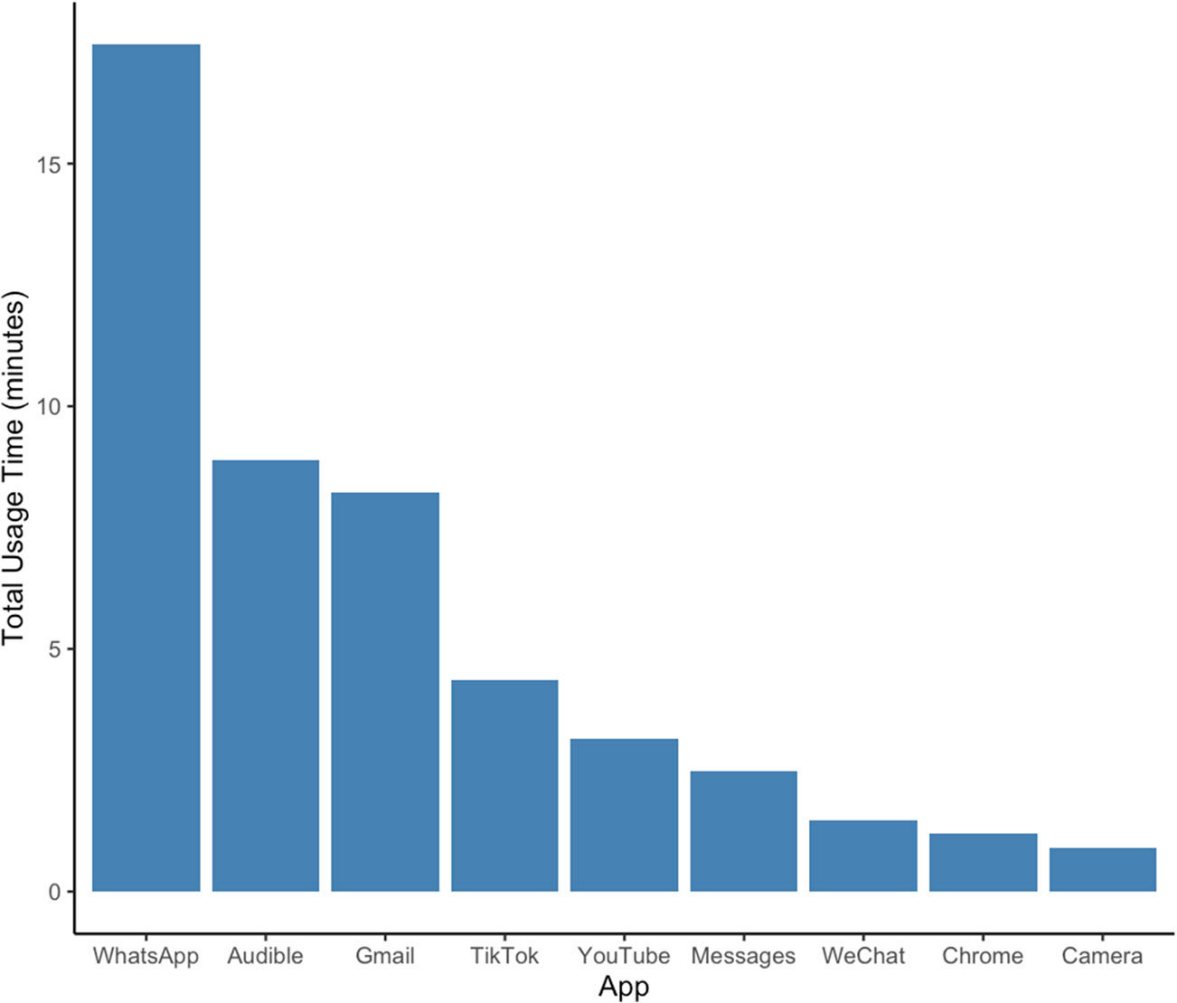
Total time spent using a selection of popular apps in a single 24-h period

**Fig. 3:**
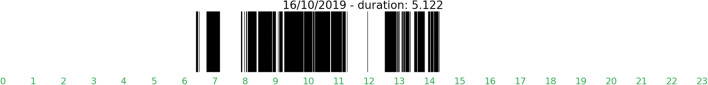
Usage of a smartphone over a 24-h period. Time and duration are reported. *Black bars* represent periods of consistent use

**Fig. 4:**
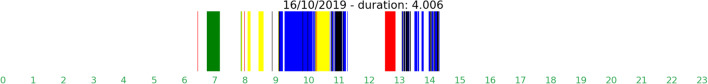
Usage of specific apps over a 24-h period. *Colors* represent different apps including: WhatsApp (*black*), Quickstep (*red*), TikTok (*green*), YouTube (*yellow*), and Google Play Store (*blue*)

Visualization provides an improved descriptive understanding of usage that has been deployed as part of previous research designs (e.g., Andrews et al., 2015; Wilcockson et al., 2018). Here, we provide code to produce simple visualizations that reflect daily and hourly periods of usage. Figure 2 illustrates how much time an individual has spent using different apps over time. Figure 3 captures how a device was used across the day alongside the first author’s ongoing battle with mild insomnia. Visualizations like this also help ensure an accurate representation of records and identify any errors. Figure 4 captures similar data showing the five most used apps, with the reported duration reflecting those specific apps. These can be customized further using the provided scripts. The potential for other visualizations remains vast particularly in terms of identifying different patterns of use at specific points in time or understanding the flow of habitual behaviors  that may be goal directed or absent minded (Marty-Dugas et al., 2018). These alone may help identify behaviors that are associated with different psychological affordances, processes, and outcomes.

## Discussion and conclusions

When it comes to understanding the impact of mass communications technology on individuals and groups, psychology’s current conclusions are only as strong as the measurements that underpin any design. The same notion applies to large swathes of research that aims to understand the predictive properties of technology use across psychological science (Ellis, 2020; Ellis et al., 2019). Existing measures have been more informed by concern around technology use (e.g., smartphone ‘addiction’) rather than making the most of technological resources at the disposal of behavioral scientists (Ellis, 2020). Several indications suggest that the relationship between smartphone usage and well-being has been overestimated when relying on objective data (Ellis et al., 2019; Katevas et al., 2018; Shaw et al., 2020).

The method documented here securely and accurately provides detailed accounts of smartphone usage. We acknowledge that further work could ensure that the security of participant data is enhanced further. For example, e-mailing a password to a researcher is only as secure as a researcher’s e-mail account. However, it is worth noting that while high-level raw data (e.g., total smartphone time) in this instance is unlikely to compromise an individual’s safety or security if shared widely, data regarding specific apps could be used to make inferences about individuals that they may wish to keep private (Ellis, 2020). Researchers should be especially mindful when linking digital traces like these with other psychological assessments or sensitive demographic variables. As a consequence, additional security procedures might include uploading data to a secure server or existing cloud service. However, this could  increase the technical threshold for adoption. The decision to make Usage Logger serverless was deliberate to ensure that researchers can use the app and comply with open science practices from the outset, but we acknowledge these benefits can generate conflicts with privacy requirements (Dennis et al., 2019). If Usage Logger was developed purely from a software security perspective, then its architecture would be very different. As it stands, security and privacy features align with the original aims by ensuring participants remain in control of their data (Dennis et al., 2019; Geyer et al., 2019). This is similar to how iPhone users can export all their Health data as an Extensible Markup Language (XML) file. However, unlike Usage Logger, these data are extensive, sensitive, and (at the time of writing) not encrypted once exported.

In many respects, Usage Logger is perhaps the start of a code base that could be diversified further in order to collect data across multiple devices and services that capture technology-related behaviors. In addition, the digital data generated from related methods have many more applications beyond those already discussed. This includes researchers going beyond device or application-level metrics. For example, Meier and Reinecke (2020) consider different types of interaction that can occur at: the device level (e.g., time spent on a smartphone), the application level (e.g., time using a specific app), or the feature level (e.g., using specific features within Twitter), which can be further segmented based on communication type. The tools reported here allow for a complete analysis at a device and application level. Some feature-level and communication inferences can be determined from an apps known functionality. Further analysis is also possible based on specific app notifications. However, while measuring technology behaviors on a more granular level can provide additional insights, technology companies will need to support comprehensive application programming interfaces (APIs) and secure access points for researchers to investigate in-app behavior (Johannes et al., 2020). Despite current limitations, Usage Logger provides access to valid and reliable data that will help researchers working across a variety of domains. This includes the ability to describe smartphone interactions and better understand their impacts. Enabling descriptive work at any level remains essential in order to aid with the development of well-grounded theory, which remains a long-standing aspiration for those studying the causal effects of new technology on people and society (Ellis, 2019; Ellis, 2020; Miller, 2012).

These developments are not to suggest that there is no place for non-behavioral measures in this domain of research. On the contrary, if a research question aims to consider a persons’ thoughts, feelings, or attitudes towards a specific technology then other measures will remain essential. However, psychometric tools to support this endeavor should be developed and used with a clear appreciation of the specific questions they can (and cannot) answer. For example, many survey instruments are not an accurate reflection of objective usage despite often being used as a proxy for behaviors (Ellis et al., 2019). Assuming that technology use is the primary variable of interest, researchers may consider moving away from latent measures completely given that ‘use’ is directly observable (Ellis, 2020).

In practice, no self-reported measure of behavior will be perfect when compared with ground truth (Orben et al., 2019). However, this ground truth is slowly becoming readily available, and we would encourage behavioral scientists to adopt these methods along side open research practices wherever possible. While not standard practice for those who often make sizable claims about the effects of technology on large swathes of the population, combing such an approach with novel analytical methods are essential for the field to progress. Only then can an interdisciplinary endeavor deliver valuable insights for both scientists and policymakers (Ellis, 2020).

## Supplementary Materials

**Links**

App - https://play.google.com/store/apps/details?id=geyerk.sensorlab.suselogger

App source code and associated websites - https://github.com/kris-geyer/UsageLoggerPublished

Psych Validator - https://github.com/kris-geyer/psychvalidaitor

Walkthrough Guide - https://u-log-walk.netlify.app/

## Footnotes

1 As a further sense-check, Shaw et al., (2020) observed similar relationships between high-level objective usage (e.g., total smartphone time) and health assessments (e.g., depression) irrespective of whether technology use was extracted from an early version of Usage Logger (N=46) or using Apple Screen Time (N=199).
